# Editorial: Unraveling pathogen-plant-microbiome interactions in horticultural crops through omics approaches

**DOI:** 10.3389/fpls.2025.1696789

**Published:** 2025-10-01

**Authors:** Abhay Kumar Pandey, Sai Shiva Krishna Prasad Vurukonda, Davide Giovanardi

**Affiliations:** ^1^ Department of Mycology & Microbiology, Tea Research Association, North Bengal Regional R & D Centre, Jalpaiguri, West Bengal, India; ^2^ Department of Engineering and Technology of Chemical Processes, Faculty of Chemistry, Wroclaw University of Science and Technology, Wrocław, Poland; ^3^ Department of Life Sciences, University of Modena and Reggio Emilia, Reggio Emilia, Italy

**Keywords:** fungal pathogens, pathogenesis, microbial diversity, virulent effectors, horticultural crops

Horticultural crops are high-value commodities with significant potential for value addition, enhancing returns for growers and supporting economic growth within agricultural production systems (Ravichandra, 2014). However, these crops face substantial threats from pests and diseases caused by bacteria, fungi, and oomycetes, which can result in considerable global yield losses and pose risks to food security (Xu et al., 2022). Pathogens utilize effector molecules—including small RNAs, proteins, and secondary metabolites such as volatile organic compounds (VOCs)—to facilitate infection (Lapin and Van den Ackerveken, 2013). Despite considerable research, the mechanisms and biological functions of effectors in model plants are not yet fully understood (Pliego et al., 2013). Comprehensive approaches are required to elucidate the complex interactions among pathogens, horticultural crops, and their associated microbiomes. Recent omics advancements have improved knowledge of the genetic and molecular strategies pathogens employ to infect hosts and evade plant immunity; nevertheless, gaps remain in understanding microbe–microbe interactions and the influence of the plant microbiome on pathogen virulence.

The Research Topic “*Unraveling Pathogen-Plant-Microbiome Interactions in Horticultural Crops Through Omics Approaches*” aimed to advance the understanding of interactions between microbial pathogens and horticultural crops using diverse omics methodologies. The objective was to provide novel insights for sustainable disease management, particularly in host resistance and breeding. This topic addressed pathogen modulation of plant-associated microbial communities, the production of volatile metabolites during infection, and coevolutionary dynamics within crops based on recent research. The call attracted significant interest, resulting in 18 accepted articles from 121 authors globally.

Jia et al. investigated the influence of microbial diversity on soil nutrient cycling in the rhizosphere of Dahongpao mother trees (*Camellia sinensis*) versus cutting-derived Dahongpao. The mother tree soils exhibited significantly higher total and available N, P, and K, as well as elevated enzymatic activities. Metagenomic analysis further revealed greater microbial diversity in mother tree rhizospheres. These results indicate that the mother tree rhizosphere harbours enriched microbial communities that enhance soil enzyme activities, nutrient transformation, and nutrient availability, thereby promoting tea plant growth.

In another study, Jia et al. investigated the diversity and functional shifts of fungal and bacterial communities in the rhizosphere soils of *Dahongpao* mother trees and cuttings, and their influence on soil nutrient transformation. Six bacterial genera and four fungal genera showed significant differences between the two rhizosphere types. Enzymatic activity and nutrient content were higher in the mother tree soil. These findings provide valuable insights for *Dahongpao* cutting cultivation, management, and microbial regulation of tea tree growth.

Zhou et al. investigated the diversity and composition of soil microbial communities in the rhizospheres of late blight-resistant and -susceptible tomato cultivars. Under natural field conditions, resistant cultivars exhibited greater bacterial diversity and abundance of specific taxa (e.g., *Azospirillum*, *Pseudomonas*, and *Acidovorax*) but reduced fungal diversity compared to susceptible cultivars. In the latter, a higher diversity of pathogenic fungi, including *Plectosphaerella* and *Neocosmospora*, was detected in susceptible rhizospheres. These findings highlight how microbial community shifts contribute to pathogen suppression in resistant tomatoes, providing valuable insights for late blight management strategies.

In northern Thailand, Suwannarach et al. identified novel *Fusarium* species (*e.g.*, *F. compactum*, *F. jinanense*, *sulawesiense*, *F. mianyangense*) associated with postharvest fruit rot of muskmelon through multi-locus phylogenetic analysis and fungicide evaluation, where copper oxychloride was found as a potential management option for muskmelon fruit rot caused by these *Fusarium* pathogens.

In another study, Kawaguchi et al. examined the population diversity of *A. vitis* across six infected vineyards in Hokkaido, Japan, a region with heavy snowfall. Bayesian modelling indicated that bacterial populations in galls were influenced by cultivar and low temperature, while those on skins were shaped by location and low temperature. Bayesian changepoint detection further revealed a winter increase in bacterial populations, particularly under snow. Populations beneath snow exceeded those above, suggesting that snow enhances bacterial survival, enabling overwintering in galls and skins and serving as inoculum for the following season.

Tenea and Molina first characterized the exocarp bacterial community structure of cape gooseberry (*Physalis peruviana* L.) and associated pathogens. Shannon diversity analysis revealed the highest bacterial diversity in unripe market fruits. *Candidatus Liberibacter* was the most frequent pathogen in field samples, while opportunistic enteric species including *Klebsiella pneumoniae*, *K. variicola*, and *Escherichia vulneris* were identified in market fruits. This study provides insight into the microbial composition of cape gooseberry exocarps along the farm-to-market chain and highlights potential food safety risks, underscoring the need for strategies to minimize bacterial contamination in both production and retail stages.

Peng et al. investigated the dynamics of phyllosphere bacterial communities in tobacco during bacterial wildfire disease, using samples collected in June (M1), July (M2), August (M3), and September (M4). Whole-genome assembly indicated that community composition was largely driven by stochastic processes. PICRUSt2 predictions showed that genes enriched in M3, coinciding with the peak disease index, were associated with virulence factor secretion, enhanced metabolic activity, and environmental adaptation. Analysis of ecological networks further revealed 139 cases of horizontal gene transfer among correlated bacterial pairs, highlighting its role in bacterial adaptability, host colonization, and pathogenicity.

A study on pink borer (*Sesamia inferens* Walker) resistance in maize reported that variation in resistance is associated with morphological traits and cell wall constituents (Soujanya et al.). Resistant and moderately resistant genotypes (CM 500, DMRE 63, and WNZ Exotic pool) showed the least leaf injury compared to susceptible genotypes (CM 202 and BML 6). Reduced injury in resistant lines was attributed to higher levels of biochemical compounds, which were found in lower amounts in susceptible lines. Resistance in maize is determined by the combined effects of multiple morphological and biochemical traits rather than a single factor, providing a basis for resistance breeding programs.

Kawaguchi investigated the infection pathways of the grapevine crown gall pathogen *Allorhizobium vitis* (= tumorigenic *Rhizobium vitis*). Over a three-year period (2020–2022), they analysed the spatiotemporal distribution of crown gall in vineyards using the binary power law model and concluded that pruning tools act as transmission vectors and that pathogen spraying contributes more to crown gall incidence than infected soil.

On anthracnose of pecan, Hu et al. used unique molecular identifier RNA sequencing (UMI RNA-seq) to provide new insights into the molecular basis of *C. fructicola* pathogenicity. Transcriptome profiling across three infection stages identified 6,822 differentially expressed genes contributing to pathogenicity. Candidate effectors Cf-ID1 and Cf-ID2, localized in the cytoplasm and nucleus, respectively, suppressed *C. fructicola* infection by triggering defense responses in *Nicotiana benthamiana*.

The RPP13 gene family plays a key role in plant resistance across various crops, but its function in potato remains largely uncharacterized. Here, Yuan et al. identified and analyzed 28 RPP13 genes associated with scab disease resistance. In particular, StRPP13–11 was significantly downregulated in both susceptible and resistant cultivars, implicating it in pathogen recognition, disease resistance and maintaining chloroplast reactive oxygen species homeostasis, offering a valuable genetic resource for potato breeding programs.

Xiao et al. applied a metagenomic approach to examine soil physicochemical properties and microbial community composition in relation to *Ralstonia solanacearum* occurrence in tobacco. They found that microbial interaction networks were more complex in the rhizosphere of healthy plants than in diseased soils. Pathogen abundance was higher in diseased soils and negatively correlated with soil phosphorus, while significantly linked to both archaeal and bacterial assemblages. Functional gene analyses within the Molecular Ecological Network revealed 468 instances of horizontal gene transfer among co-occurring bacterial taxa. The findings highlight the importance of soil microbial interactions and horizontal gene transfer in disease suppression and suggest that sustainable management strategies should integrate microbial community functions to control tobacco bacterial wilt.

Tagyan et al. modelled the global distribution of *R. solanacearum* under current and future climate scenarios, identifying annual mean temperature as the primary environmental predictor of its distribution in crops. They emphasized the urgent need for sustainable agricultural measures and the development of novel, eco-friendly strategies to control *R. solanacearum* spread, especially in developing regions, to support food security amid climate change. In potato, Lai et al. reported that *R. solanacearum* infection caused spatial shifts in the potato root-associated microbiome and metabolome. Healthy plants harboured higher microbial populations compared to diseased ones, highlighting the potential of microbiome engineering strategies for bacterial wilt management.

In smallholder farming systems of western Kenya, Mutai et al. evaluated simple bioassays based on morphological features, pathogenicity, and sequencing for soil-borne pathogens, including *Fusarium*, *Pythium*, and plant-parasitic nematodes. Regarding management, soils amended with manure showed lower populations of these pathogens and nematode, along with increased beneficial microbes, compared to soils amended with synthetic N and P fertilizers. These findings indicate that low-cost bioassays can aid timely detection of soil-borne pathogens, supporting informed decisions on soil health and pathogen management.

Giovanardi et al. reviewed biocontrol strategies, such as microbial biocontrol agents, botanicals and bacteriophages, against bacterial diseases in tomato and other vegetables including bean, pepper, cabbage, and cauliflower. While such approaches can reduce reliance on synthetic pesticides and enhance crop productivity, their large-scale application remains constrained by regulatory variability, compatibility issues among microbial agents, and inconsistent field performance.

Myxomycetes, or plasmodial slime molds, are associated with several diseases in crops and cultivated mushrooms. Zhang et al. reviewed diverse myxomycete species implicated in such diseases. This review highlighted the economic importance, taxonomic diversity, and microbial interactions involved in myxomycete colonization, as well as available cultural and chemical control strategies. Evidence indicates that while myxomycetes are not recognized as plant pathogens, certain species significantly affect global mushroom production, underscoring the need for effective management approaches.

The genus *Colletotrichum* causes anthracnose in numerous crops and is responsible for substantial pre- and post-harvest losses worldwide. Salotti et al. developed a predictive model for anthracnose epidemics based on literature covering *Colletotrichum*–host interactions. The model estimates disease progress on foliage during the growing season. *Colletotrichum* species were classified into seven clades. The model was tested for anthracnose outbreaks caused by five of these groups across six host species, using data from 17 epidemics in the USA, Italy, Canada, and Japan. Following large-scale validation, this model could assist in decision-making for anthracnose management.

Contributions to this Research Topic emphasize the multifaceted colonization strategies of pathogens in horticultural and other crops. The scope includes disease prediction models, identification of virulent agents, elucidation of pathogenic and resistance mechanisms through omics approaches, and strategies for disease prevention under climate change.
